# Neuroprotective Role of Phytochemicals

**DOI:** 10.3390/molecules23102485

**Published:** 2018-09-27

**Authors:** Bharath Kumar Velmurugan, Baskaran Rathinasamy, Bharathi Priya Lohanathan, Varadharajan Thiyagarajan, Ching-Feng Weng

**Affiliations:** 1 Toxicology and Biomedicine Research Group, Faculty of Applied Sciences, Ton Duc Thang University, Ho Chi Minh City 700000, Vietnam; 2 Graduate Institute of Anatomy and Cell Biology, College of Medicine, National Taiwan University, Taipei 10617, Taiwan; rathinabaski@gmail.com; 3 Division of Radiation Oncology, Department of Oncology, National Taiwan University Hospital, Taipei 10617, Taiwan; bharathi.log@gmail.com; 4 Department of Pathology, Anatomy and Cell biology, Thomas Jefferson University, Philadelphia, PA 19107, USA; varadha86@gmail.com; 5 Department of Life Science and Institute of Biotechnology, National Dong Hwa University, Hualien 97401, Taiwan

**Keywords:** phytochemicals, neurogenerative disease, curcumin, EGCG, resveratrol, limonoids

## Abstract

Neurodegenerative diseases are normally distinguished as disorders with loss of neurons. Various compounds are being tested to treat neurodegenerative diseases (NDs) but they possess solitary symptomatic advantages with numerous side effects. Accumulative studies have been conducted to validate the benefit of phytochemicals to treat neurodegenerative diseases including Alzheimer’s disease (AD) and Parkinson’s disease (PD). In this present review we explored the potential efficacy of phytochemicals such as epigallocatechin-3-galate, berberin, curcumin, resveratrol, quercetin and limonoids against the most common NDs, including Alzheimer’s disease (AD) and Parkinson’s disease (PD). The beneficial potentials of these phytochemicals have been demonstrated by evidence-based but more extensive investigation needs to be conducted for reducing the progression of AD and PD.

## 1. Introduction

Neurodegenerative diseases (NDs) have become a menace in the 21st century and currently there is no effective treatment to cure these types of diseases. Neurodegenerative diseases are more prevalent in the old age population. Occurrence of NDs is increasing all over the word due to increased proportion of old age population. Development of NDs in old age people is systematic and finally leads to death. The most common NDs observed in elderly people are Alzheimer’s disease (AD) and Parkinson’s disease (PD), in which dementia is associated with AD and movement disorder in patients with PD.

**Figure 1:**
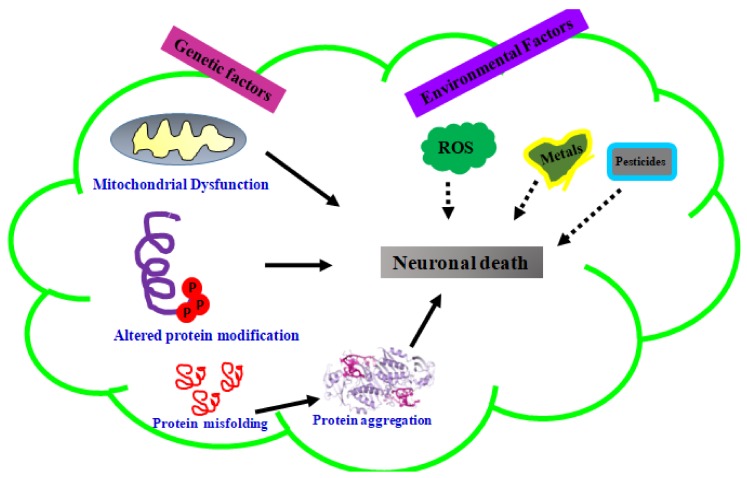
Various factors involved in progression of neurodegenerative disease.

**Figure 2:**
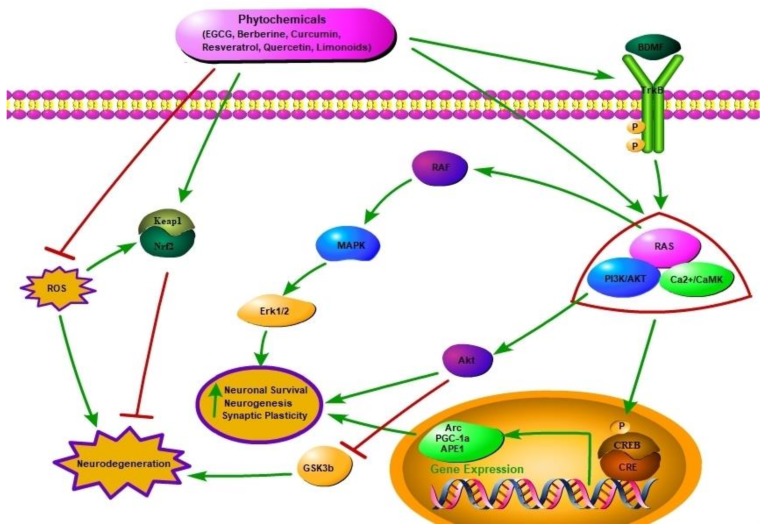
Two sites action of phytochemicals on alleviating progression of neurodegenerative diseases. Activation of TrkB receptor by BDNF or Phytochemicals induces PI3K/Akt kinase signalling and Ras, Raf and ERK pathways resulting in protecting neurons from degeneration. BDNF activates transcription factor-CREB which further induces the expression of Arc (synaptic plasticity), PGC-1α (cellular energy metabolism) and APE1 (DNA repair enzyme).

Neurodegenerative diseases such as AD, PD, Huntington’s disease (HD), and multiple sclerosis (MS) fall under the umbrella of neurological disorders along with neurotraumatic disorders [1,2]. Genetic and environmental factors also play an important role in the progression of neurodegenerative diseases (Figure 1). Common pathological features of these neurodegenerative diseases are oxidative stress, accumulation of certain aggregated proteins and neuroinflammation [3]. These stimuli through extracellular receptors embedded in the plasma membrane, and neurotrophins causes cellular stress and induce production and release of brain-derived neurotrophic factor (BDNF), and activation of tropomyosin-related kinase (TrkB) receptor family and other downstream protein kinases (Figure 2) [4,5]. Neurotrophins are essential for the survival, maintenance, and regeneration of specific neuronal populations in the brain [6]. Neurotrophin expression has a strong correlation with neurodegenerative disease occurrence [7]. Mammalian neurotrophins, such as nerve growth factor (NGF), brain-derived neurotrophic factor (BDNF), neurotrophin-3 (NT-3), and neurotrophin-4/5 (NT-4/5) are found to play an vital role in maintenance of neurons in CNS and PNS [8]. Neurotrophins were properly recognized as promoters of neuron survival and various bodies of evidence have been suggested that NDs are caused via rampant decline in neurotrophin levels [9]. Thereby, neurotrophins have become important targets for phytochemicals against ND. The mechanism by which neurotrophins prevent neuron degeneration is by binding and activating the Trk receptor family which is present in the plasma membrane. The neurotrophins bind to Trk receptors and consequently constitute a growth-promoting microenvironment for neurons [10]. Once this binding occurs, activation of various intracellular signaling pathways like ERK and PI3k/AKT which promote cell survival and help in rescue of neurons from neurodegeneration happens. In addition to signaling pathway activation, neurotrophin also promotes Bcl-2 gene expression, which acts as an intracellular apoptosis inhibitor. There is gradual degeneration of neurons in the absence of binding of neurotrophins to the Trk receptor family. Combating neurodegeneration by administrating neurotrophins is an effective way to overcome this disease; however getting across the blood brain barrier (BBB) remains one the greatest challenge to treat central nervous system disorders.

Different symptoms and neurological signs such as dementia and motor disorders are observed in the patients with NDs. Interestingly, most of these signs and symptoms are observed to be common for the NDs like AD, PD and HD [11]. Early detection of neurodegeneration can provide a chance for an early treatment that may be helpful to prevent further progression of the disease [12,13,14]. Inhibition of NMDA receptor can prevent/delay AD; memantine and namzaric are the drugs used for treating AD patients, which act as an antagonist for NMDA receptor [15]. Recently, Axona is used for AD treatment, which acts as an alternative energy source [16]. Various drugs are used for treating PD, including apomorphine hydrochloride, rotigotine, ropinirole and levodopa [17]. Levopoda prevents PD symptoms mainly associated to bradykinesia [18]. Tolcapone, catechol-*O*-methyl-transferase inhibitor treatment along with levopoda can increase the levopoda concentration in plasma [19]. Rotigotine is recommended for advanced stages of PD [20], whereas ropinirole is used for early stages [21]. Current treatment strategies for NDs are only associated with symptomatic relief. They can only get rid of positive and negative symptoms developed owing to neurodegeneration rather than curbing their progression. Although, drugs are available in treating the symptoms associated with the ND, therapeutics or preventive medicines for curing/preventing neurodegeneration are not available [22]. so the problem of how to treat these diseases still persists [23].

**Figure 3:**
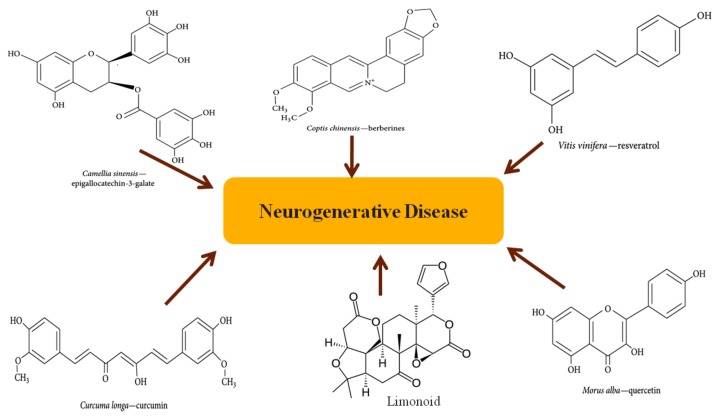
Structures of representative phytochemicals (epigallocatechin-3-galate, berberin, curcumin, resveratrol, quercetin, limonoids).

Thus it is necessary to develop new and more effective therapeutic strategies to combat these devastating diseases. Fighting chronic disease by phytochemicals or herbal medicine has become a hot topic recently, and numerous studies using phytochemicals for treating NDs have been published [6]. This review emphasizes the importance of phytochemicals (Figure 3) in Alzheimer’s disease (AD) and Parkinson’s disease (PD), in particular the potential mechanism of action of these natural compounds.

## 2. Overview of Phytochemicals

Nowadays, there is pressing need for daily consumption of fruits and vegetables because they are full of phytochemicals [24]. Phytochemicals are naturally found and they are shown to have protective action against oxidative stress and neuroinflammation, which are major hallmarks of NDs [25]. Common phytochemicals which we come across in our daily lives are curcumin, quercetin, diallyl trisulfide, flavonoids and epigallocatechin-3-galate (EGCG). Phytochemicals induces immune system, decreases platelet aggregationand regulates hormone metabolism [3].

One group of researchers has proposed that greater consumption of olive oil-containing Mediterranean diet results in a significant improvement in health status, as seen by a significant reduction in overall mortality (13%) in PD and AD patients [26]. Cumulative evidences have shown that phytochemicals as nutraceuticals can alleviate the condition of NDs but the underlying mechanism is completely unknown. One study has claimed that phytochemicals improve the condition by their anti-oxidative and radical scavenging capacity [27]. Cellular targets of phytochemicals are not known, but it is hypothesized that these compounds cannot perform their function by only regulating enzyme metabolism and gene expression, and they might be activating stress response pathways which the cells will use as a shield [28]. Another hypothesis is that phytochemicals act as a ligand, binding to particular receptors on cell membranes or nuclei, subsequently participating in a downward signal transduction pathway and exhibiting their anti-oxidant effect [29].

### 2.1. Neuroprotective Potential of Phytochemicals

#### 2.1.1. Epigallocatechin-3-Galate

The most widely found polyphenol in tea leaves is epigallocatechin-3-galate (EGCG) and in the past few decades, it has caught the eye of everyone due to its potential in delaying the degeneration of neurons. The incidence of NDs is inversely correlated with tea consumption [30]. Many research studies have been carried out in AD models where EGCG was administered. In one of these studies, a drug called D-gal was administered to AD models and it drastically reduced amyloid plaques [31,32,33]. Another research group has suggested that by administering EGCG, β- and γ-secretases were decreased by inhibiting ERK and NF-κβ, thus preventing neuronal cell death [34]. Another study suggested that by administering EGCG, amyloid plaques were reduced due to cleavage of APP by α-secretase [35]. In the case of PD, one study has suggested that adults who had three or more cups of tea faced decreased risk of developing PD. It also found that the free radical scavenging system is strengthened by increasing glutathione which in turn activates CREB and Bcl-2 which leads to positive manifestations [36]. In PD, there is severe loss of tyrosine hydroxylase (TH) positive cells and so one study has suggested that simultaneous intake of tea and EGCG prevented the loss of these cells in substantia nigra [37]. All these results indicate that EGCG can be used as a care treatment for ND. The molecular mechanism which EGCG inhibits neuronal cell death is by stopping dopamine (DA) uptake and exerting neuroprotection. It carries out this function by acting on the enzyme catechol-*O*-methyltransferase (COMT) which blocks the metabolism of DA. In addition to it, α-synuclein aggregates are found in ND and EGCG is thought to regulate proteolytic cleavage of α-synuclein converting it into a less toxic form [34,37].

On the other hand, the action of EGCG is concentration-dependent. Low concentrations of EGCG exert neuroprotection while high concentrations of EGCG tend to have anti-proliferative effects and prevent angiogenesis. [37]. EGCG is an important member of the catechol family with a 3,4,5 trihydroxy B ring, due to which it exhibits anti-oxidant effects and nullifies radicals [38]. The pathway through which EGCG exhibits anti-oxidant properties is the Nrf2/ARE system [39]. In one study, it has been found that it activates two radical scavenging enzymes, SOD and catalase [38,40].

The reason why EGCG offers a promising solution is because of its ability to cross the BBB and its metal chelating properties. It has been observed in one study that EGCG prevents iron-dependent α-synuclein aggregation and restores DA neurons [41]. After conducting many clinical studies, the fact has been established that it inhibits cytochrome p450 and is responsible for lipid peroxidation in vitro as well as in vivo [42,43]. At low concentrations, it prevents oxidation while at higher concentrations it exhibits pro-oxidant properties [44].

In SH-SY5Y cells, EGCG exhibits neuroprotection by playing a critical role in the intracellular signaling pathway of protein kinase C (PKC) and it is observed that PKC inhibitors participate in the mode of neuroprotection by EGCG. EGCG inhibits the neurotoxin 6-hydroxydopamine (6-OHDA) by activating phosphorylation of protein kinase C (PKC) [37]. Not only the PKC signaling pathway, but other pathways like PI3k/Akt, MAPK, and pathways regulating calcium influx are also activated [45,46]. In one study, it has been observed that EGCG does not phosphorylate ERK1/ERK2 but it participates in a decrease in ERK1/2 levels caused due to oxidative stress [47].

There is close relationship between inflammation and neurodegenerative diseases and EGCG reduces inflammation by reducing the expression of inflammatory molecules in IL1β, TNF and TGB [48,49]. However, contradictory results were observed when high concentrations of EGCG were administered. At higher concentrations, it increases expression of the inflammatory molecules TNF and IL6 as compared to a lower dose at which it offers neuroprotection. The exact mechanism of how EGCG exerts anti-inflammatory properties thus is still unknown [50].

#### 2.1.2. Berberine

Various studies in neurodegenerative models have suggested that berberine confers neuroprotection by regulating neurotrophin levels [51,52]. It has also been described that berberine is able to stop the activity of various enzymes involved in the progression of AD [53]. Various in vitro studies were carried out in which neurotoxicity was induced through glutamate, H_2_O_2_ and a reduced oxygen environment created by cobalt chloride. In these in vitro studies, it was confirmed that berberine is able to save neuronal cells from neurotoxicity [53,54,55]. Berberine has also been found to be useful in the treatment of hypoxia induced by cobalt chloride. In this particular condition, it acts as a scavenger of reactive oxygen species (ROS) generated due to the hypoxic conditions, which in turn suppresses various apoptosis-promoting agents, thus conferring neuroprotection [56]. However, Kwon et al. have reported that berberine has profound side effects, which include a decline in DA neurons due to the cytotoxicity of 6-hydroxydopamine [57].

Berberine exhibits neuroprotective effects by activation of the PI3K/Akt/Nrf2 pathway by scavenging radicals. Moreover, it has been observed that berberine exhibits anti-apoptotic effects by reducing the expression of caspase 1 and 3, bax as well as upregulation of Bcl-2 [58]. One study by Hsu et al. has shown that berberine administration prevents hydrogen peroxide-induced neurotoxicity by reducing the expression of p53, caspase, cyclin D1 and increasing the expression of Bad [59]. It has been shown that berberine when administered at nanomolar concentration it promotes cell survival and reduces oxidative stress by downregulating various factors such as cytochrome c, Bax and caspase. In the case of ischemic stroke, it has been observed that administration of berberine can protect by inhibiting potassium currents [60].

In other clinical study, it has been observed that berberine administration reduces superoxide dismutase and cholineacetyl transferase in neurodegenerative animal models [58]. However, many studies have also shown that berberine, when administrated for longer periods, is responsible for loss of dopaminergic neurons in substantia nigra [57,61,62]. It has been suggested that berberine protects against ischemic stroke by scavenging radical species, or in other words, reducing oxidative stress [63]. In an AD rat model, administration of berberin restores memory function and levels of brain-derived neurotrophic factor, takes care of cholinergic enzyme, and decreases the expression of inflammatory molecules [52]. There is one suggestion that berberine works through the NMDA receptor and confers neuroprotection. In this study, berberine exceeding 1 μM reduced neuronal viability in a caspase-independent manner by early alterations of mitochondrial function and morphology. Inhibition of NMDA receptors by memantine and MK-801 completely blocked berberine-induced neurotoxicity [64]. Berberine also exhibits anti-apoptotic properties by decreasing the expression of p53 and HIF-1α which are thought to be main mechanisms to prevent apoptosis by administration of berberine [56]. Berberine is thought to alleviate the damages caused by the ischemic stroke by reduced expression of NeuN, laminin, MMP 9 and gelatinase activity [65]. Berberine is thought to have promising role as therapeutics for Alzheimer’s disease because it can solubilize β-amyloid plaques in mice models [58].

#### 2.1.3. Curcumin

Curcumin is one of the important constituents of turmeric, which holds a prominent place among Indian spices. Curcumin has various medicinal properties and therefore it is used in the treatment of diabetes, biliary disorders, cough and hepatic disorders [66,67,68,69]. One of the mechanisms that has been suggested for curcumin is that it has ability to bind to amyloid plaques by inhibiting NF-κβ thus reducing the pathogenesis of AD [66]. This was checked in an experiment in which around 214 compounds having anti-oxidant properties were checked and curcumin was found to have the highest affinity towards amyloid plaques [70]. Cognitive impairments were improved in aged mice due to an increase in CREB and BDNF levels caused by administration of curcumin [66]. It has also been shown that curcumin helps in regenerating neurons by activating Trk/PI3K signaling pathways which elevate BDNF levels in a PD model [71]. Curcumin is thought to function by reducing TNF-α and caspase levels and simultaneously elevating BDNF levels [66,72]. In recent studies, curcumin nanoparticles have also been prepared, which alleviate the condition of cognitive impairment by recovering BDNF levels via Akt/GSK-3β signaling pathways [73]. Curcumin offers a promising safe and cheap preventive measure for ND because it acts on various molecular aspects of these diseases.

The current research focuses on finding of curcumin derivatives that will have more lipophilic properties to help them cross the BBB and will have more affinity for amyloid plaques [74]. Recent research has also shown that curcumin reduced amyloid plague formation in vivo and in cell cultures [75]. In one study conducted by Zhang et al., it was shown that curcumin not only disrupts Aβ levels but it also prevents maturation of amyloid-β precursor protein (APP) in mouse neurons [76]. The mechanism through which curcumin protects from Aβ plaques is by inhibition of NF-κB. Not only is curcumin active against AD, but curcumin also able to protect from PD by destabilization of α-synuclein protein [72]. 

Inflammation worsens the case in case of NDs and curcumin tends to reduce the expression of IL-6 and TNF-α. It has been found that brain macrophages loses their functionality in AD and cannot phagocytose properly [77]. Surprisingly, it has been found that curcumin restores phagocytosis and was able to clear amyloid plaques in the microglial cell line N9 [78]. In research studies, it has been found that amyloid plaques in AD patients contain iron and their presence is also responsible for generating advanced glycation end-products (AGEs), free radicals, oxidation of proteins and DNA, lipid peroxidation, etc. and in vitro studies have shown that curcumin shows affinity for this metal [72]. PD models can be generated by 6-OHDA and 1-methyl-4-phenyl-1,2,3,6-tetrahydropyridine (MPTP) and curcumin alleviates conditions in these models by inhibition of ROS [71,79]. It is seen that curcumin inhibits the JNK pathway in MPTP- and MPP-induced neurotoxicity in animal models [80]. The effect of curcumin has been studied in fruit fly models and it has been shown that curcumin improves lifespan and locomotion in different fruit fly AD models [81]. 

In the case of mammals, Aβ-expressing mice were prepared and curcumin was administered to these mice [82]. It was found that curcumin alleviated ND by scavenging ROS, disrupts amyloid plaques, exhibits anti-inflammatory and anti-apoptotic effects [83]. Recently, one study has found that curcumin can prevent aggregation of Huntington protein in an animal model when administered at a concentration of 500 ppm [84]. Curcumin has been found to impart many benefits in vitro and it was shown to significantly improve the lifespan in Drosophila and *C. elegans* but it shows no improvement in mice [85,86]. One of the main problems associated with curcumin is its poor bioavailability, which could be addressed by changing the chemical structure of curcumin or by conjugation of curcumin with lipophilic compounds [87].

#### 2.1.4. Resveratrol

It is a phenolic compound which is found in grapes, peanuts, wine and tea. It is called a “miracle” molecule because it exhibits antioxidant and anti-inflammatory properties with regards to ND [88]. Various cognitive defects that were induced in rat models were reversed following the treatment of resveratrol by inhibiting TNF-α and IL-1β levels and elevating BDNF levels in the hippocampus [88]. Further studies indicated that resveratrol increases IL-10 levels which promotes anti-inflammation by inhibiting TNF-α and NF-κβ levels. ERK1-2/CREB signaling pathways were activated, which promotes the survival of neurons by increasing the secretion of BDNF and GDNF levels after administration of resveratrol [88,89].

During ND, glial cells are activated releasing inflammatory cytokines and various neurotoxic molecules such as nitric oxide, superoxide, etc. Resveratrol confers neuroprotection by suppressing the activation of glial cells [89]. In one study, it has been found that in the model prepared by administering streptozotocin, resveratrol succeeds in restoring cognitive functions [90]. Resveratrol is found to have a role in destabilizing plaques and it prevents the formation of plaques [91]. In one recent study, it was observed that resveratrol prevents neurotoxicity caused by 6-OHDA by activation of SIRT-1 which was lost in the presence of inhibitor of SIRT1 i.e., sirtinol [92]. This is because resveratrol and SIRT-1 are both important to prevent neuronal death and resveratrol performs its function by preventing deacetylation of substrates of SIRT 1 i.e., p53 and PGC-1α [93]. Resveratrol displays neuroprotective effects in rotenone-induced PD cellular models through activation of the AMPK-SIRT1-autophagy pathway [94].

The anti-inflammatory and anti-oxidative properties of resveratrol have been thoroughly studied since last few years [95,96,97,98], but recently the activation of SIRT-1 and vitagenes by resveratrol has caught fire [93]. One study has shown that resveratrol works in the same way as calorie restriction works in increasing longevity [99]. The molecular mechanism of resveratrol is by deacetylation of PGC-1α and activation of PPAR [100]. The activation of PPAR leads to activation of catalase gene through PI3K/Nrf/keap pathway [101]. Resveratrol offers neuroprotective effects by inducing mitochondrial biogenesis by activation of AMPK [94]. Reactive oxygen species are identified as one of the major causes of neurodegeneration, which is caused to NADPH oxidase and mitochondrial dysfunction [102]. All these studies demonstrate that resveratrol offers a promising solution for NDs.

#### 2.1.5. Quercetin

Flavanoids are found in various fruits and vegetables and constitute a large heterogenic group of benzo-*ç*-pyrone derivatives. It has been reported that phenolic phytochemicals protect from complicated diseases like cancer, hepatic disorder and NDs [103,104,105]. Flavanoids are widely studied because of their anti-oxidant and free radical scavenging properties and quercetin is being widely studied for anti-proliferative capacity [106]. Cell viability was significantly decreased when phenochromocytoma cells (PC12) were incubated with hydrogen peroxide, but when PC12 were reincubated with quercetin, it protected the cells from H_2_O_2_-induced toxicity although quercetin acted in a dose-dependent manner [107]. Neuroprotective effects of quercetin were observed at a lowest concentration of 10 µM and at a highest of 30 µM. It has also been reported that quercetin can cross the BBB and prevents cytotoxicity induced by H_2_O_2_ [107]. Activity of kinases is regulated by quercetin which in turn modulates cellular function and gene expression. It has been demonstrated that the properties of quercetin depend on the arrangement of functional groups quercetin only has one disposition – if different it is not quercetin anymore [108]. The effect of quercetin depends on the cell type and exposure time and concentrations higher than 100 µM exhibited apoptotic, anti-proliferative, cytotoxic and genotoxic activities. NF-κB is found to be regulated by quercetin which could lead to improvement in inflammatory processes involved in NDs [109]. It has also been reported that protein kinases and lipid kinases such as phosphoinositide 3-kinase (PI3-kinase), Akt/PKB, tyrosine kinases, protein kinase C (PKC), and MAP kinases were also modulated by quercetin [109]. This is helpful because it changes the phosphorylation state of the target molecules thus deciding the cellular fate. It has been reported that quercetin stimulates the activation of pro-apoptotic and potentially anti-apoptotic pathways [110]. It has also been shown that biogenesis of mitochondria is enhanced by quercetin and this is important because mitochondrial dysfunction leads to neuronal degeneration by depletion of cellular ATP levels and ROS generation. Thus, quercetin protects from neurodegeneration by mitochondria targeted effects [109]. However, the absorption and metabolism steps after quercetin uptake raise many questions as to whether quercetin can be applicable to in vivo neuroprotection and much research is needed to establish its potential use in acute neurodegeneration conditions [111].

#### 2.1.6. Limonoids

Limonoids are highly oxygenated compounds and sparsely distributed in the plant kingdom. *Melia toosendan*, of the family Meliaceae, is bitter owing to an abundance of limonoids and the extract of this plant induces neuronal growth similar to NGF which functions through ERK and protein kinase A (PKA) [112]. The three limonoid compounds (dregeanin DM4, rohituka 3 and trichilia lactone D5) studied in this work were isolated from seeds of *Trichilia welwitschii* [113].

In one study, it has been found that limonoids promote neuronal differentiation and growth by increasing NGF levels in a PC12 rat cell line [112]. Extract of the plant *Melia toosendan* contains limonoid compounds which promote neurite growth similar to NGF [114]. However, it was also found in this study that when the PC12 rat cell line was exposed to PKA inhibitors, it hampers the pathway involved in neuronal growth like the PKA and ERK pathways [115]. Limonoids promote neuronal differentiation through activation of ERKs and PKA [112]. The extract of *Melia toosendan* was more successful in promoting growth as compared to that of NGF. However, in that study it was found that extract of this plant did not activate Ras/Raf1 but it is dependent on PKA/MEK activation [115]. In PC12 differentiation, p38 MAPK plays an important role but when it was inhibited, it did not affect the action of *Melia toosendan* extract [115]. PKA gets activated by accumulation of cAMP but activation of PKA by limonoids does not depend on accumulation of cAMP [116]. However, there might be a case where *Melia toosendan* extract might contain ingredients which act like cAMP and cAMP that might be accumulating at low levels and are not detectable [115]. Further investigations are needed to further demonstrate the up-regulation or down-regulation in the expression of certain genes following administration of limonoid compounds.

## 3. Conclusions

This review discusses some of the most commonly used naturally available phytochemicals that can be used to treat neurodegenerative diseases. These phytochemicals protect against neuronal damage and this review explains various pathways through which these phytochemicals protect against NDs. The potential benefits of these phytochemicals have been well studied, but more extensive studies need to be conducted in order to establish the long term effects and efficacy of using phytochemicals as therapeutics for neurodegenerative diseases.
